# Global Diversity of Brittle Stars (Echinodermata: Ophiuroidea)

**DOI:** 10.1371/journal.pone.0031940

**Published:** 2012-03-02

**Authors:** Sabine Stöhr, Timothy D. O'Hara, Ben Thuy

**Affiliations:** 1 Department of Invertebrate Zoology, Swedish Museum of Natural History, Stockholm, Sweden; 2 Museum Victoria, Melbourne, Victoria, Australia; 3 Department of Geobiology, Geoscience Centre, University of Göttingen, Göttingen, Germany; Ecole Normale Supérieure de Lyon, France

## Abstract

This review presents a comprehensive overview of the current status regarding the global diversity of the echinoderm class Ophiuroidea, focussing on taxonomy and distribution patterns, with brief introduction to their anatomy, biology, phylogeny, and palaeontological history. A glossary of terms is provided. Species names and taxonomic decisions have been extracted from the literature and compiled in The World Ophiuroidea Database, part of the World Register of Marine Species (WoRMS). Ophiuroidea, with 2064 known species, are the largest class of Echinodermata. A table presents 16 families with numbers of genera and species. The largest are Amphiuridae (467), Ophiuridae (344 species) and Ophiacanthidae (319 species). A biogeographic analysis for all world oceans and all accepted species was performed, based on published distribution records. Approximately similar numbers of species were recorded from the shelf (n = 1313) and bathyal depth strata (1297). The Indo-Pacific region had the highest species richness overall (825 species) and at all depths. Adjacent regions were also relatively species rich, including the North Pacific (398), South Pacific (355) and Indian (316) due to the presence of many Indo-Pacific species that partially extended into these regions. A secondary region of enhanced species richness was found in the West Atlantic (335). Regions of relatively low species richness include the Arctic (73 species), East Atlantic (118), South America (124) and Antarctic (126).

## Introduction

### General background

**Figure 1:**
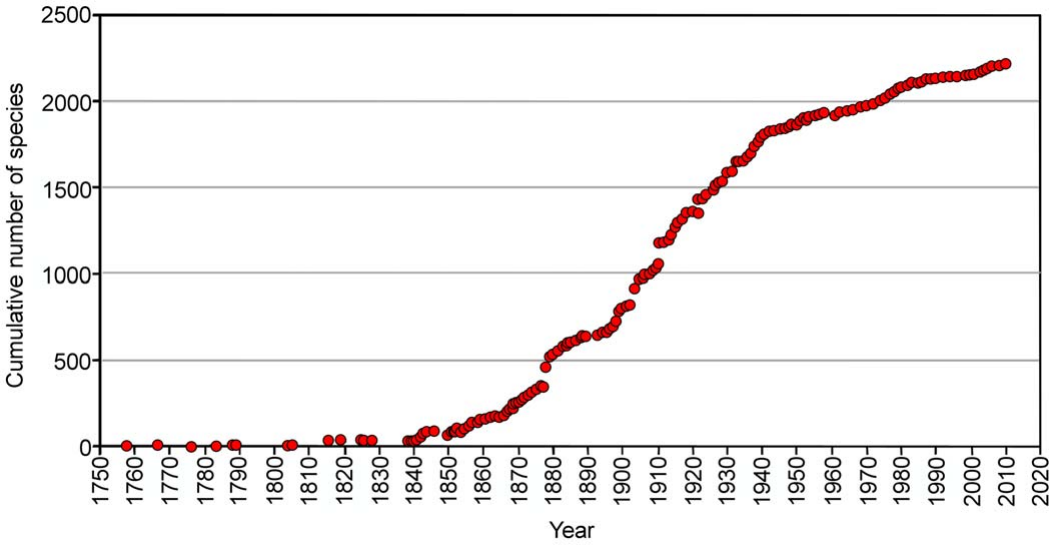
Discovery rate of ophiuroid species since 1758.

The Ophiuroidea or brittle stars, basket stars (euryalids with branching arms) and snake stars (euryalids with non-branching arms), are the largest group among extant echinoderms, with 2064 described species [1], found in all oceans from the intertidal to the greatest depths. The name Ophiuroidea is derived from the Greek words *ophis*, meaning snake, and *oura*, meaning tail, in reference to the often thin, snail-like winding or coiling arms. The discovery of the currently recognized extant species began with two descriptions, published in the Systema Naturae [2] (*Asterias caput-medusae* Linnaeus, 1758), now in *Gorgonocephalus,* and *Asterias ophiura* Linnaeus, 1758, now in *Ophiura*). From the mid-eighteenth century, the discovery rate accelerated and remained relatively high for about a century, when it levelled-off to today's lower rate (Fig. 1). Remarkably, the first deep-sea animal ever to be reported on was the brittle star *Gorgonocephalus caputmedusae* accidentally dredged up by Sir John Ross in 1818 while sounding the bottom of Baffin Bay in his attempt to find the North West passage [3]. The first fossil ophiuroid was described as early as 1804 from the Middle Triassic of Göttingen, Germany [4] (*Asterites scutellatus* Blumenbach, 1804; now in *Aspiduriella*). The description rate for fossils has remained relatively low and constant since that date. The use of isolated skeletal elements (see glossary below) as the taxonomic basis for ophiuroid palaeontology was systematically introduced in the early 1960s [5] and initiated a major increase in discoveries as it allowed for complete assemblages instead of occasional findings to be assessed.

This review provides an overview of global ophiuroid diversity and distribution, including evolutionary and taxonomic history. It was prompted by the near completion of the World Register of Marine Species (http://www.marinespecies.org) [6], of which the World Ophiuroidea Database (http://www.marinespecies.org/ophiuroidea/index.php) is a part. A brief overview of ophiuroid anatomy and biology will be followed by a systematic and biogeographic synthesis.

### Anatomy

**Figure 2:**
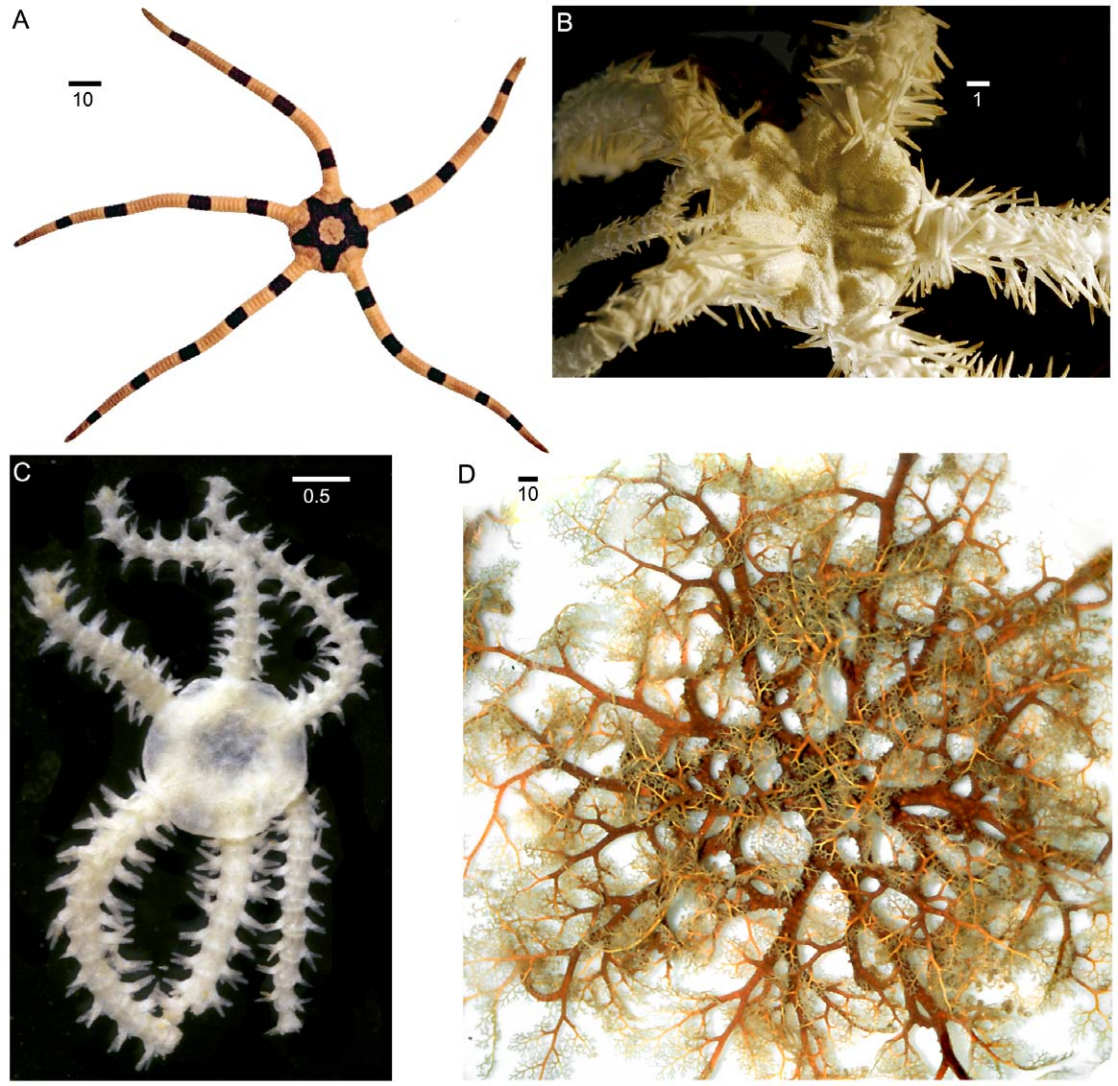
Diversity of brittle stars. A. *Ophiolepis superba*, a typical five-armed form with simple arms; B. *Ophiacantha enopla veterna*, a form with long serrated arm spines and spinelets covering the disc; C, *Ophiactis tyleri*, a six-armed fissiparous form; D. *Euryale aspera*, a basket star with branched arms. Scale bars in millimetres.

The typical ophiuroid body plan shows a pentagonal to round central disc that is offset clearly from the five arms; but a considerable number of species depart from this generalized shape. Species with six, seven and up to ten arms are known. In basket stars the arms branch once or multiple times (Fig. 2). Most species are moderate in size with disc diameters between 3 mm and 50 mm; the largest species of basket stars may have discs of 150 mm diameter. The length of their arms is usually measured in relation to their disc diameter and varies from about 2–3 times the disc diameter to 20 times or more (e.g. *Macrophiothrix*, *Amphiodia*).

**Figure 3:**
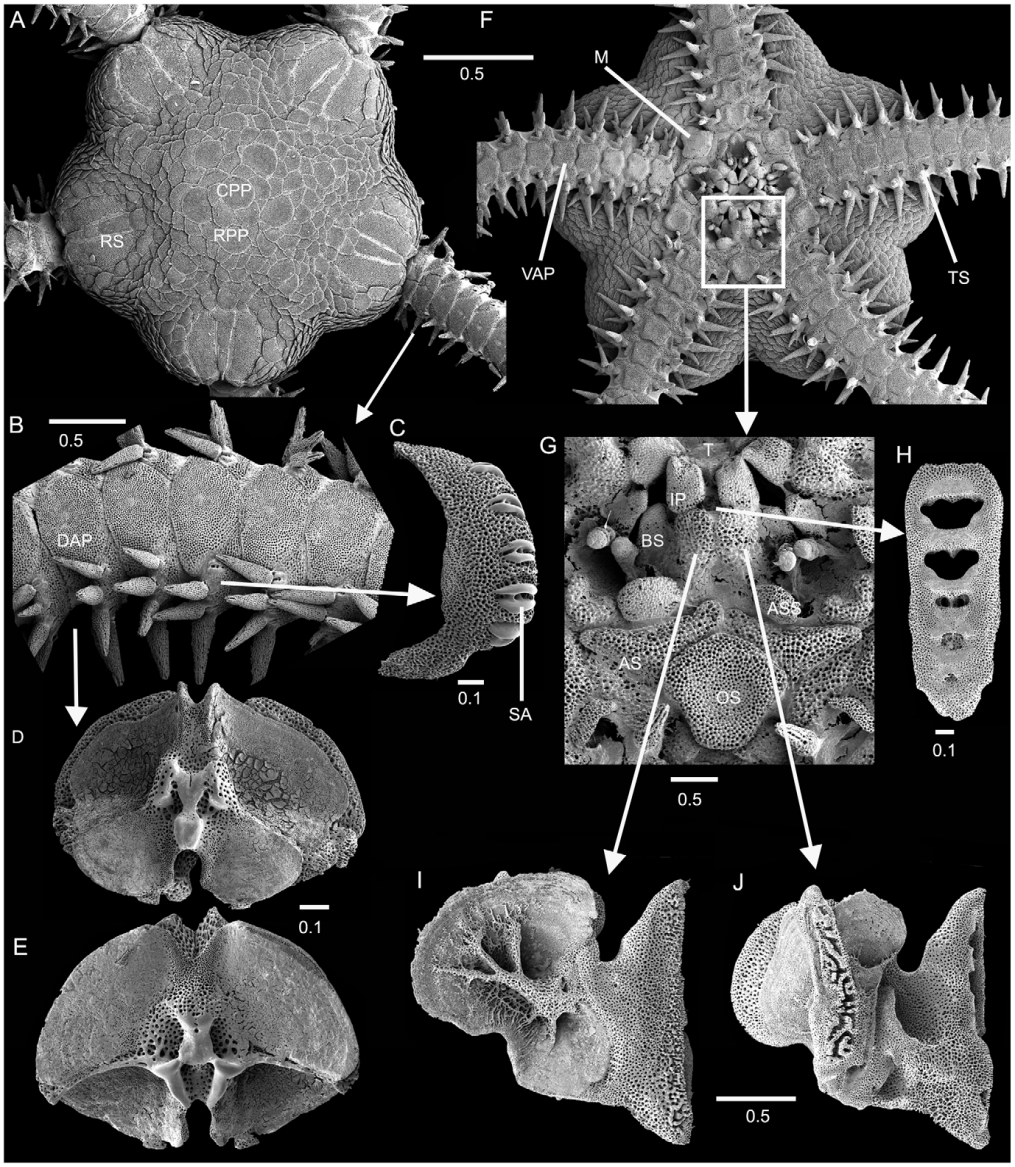
Skeletal morphology of brittle stars shown on *Amphiura chiajei*. SEM images. A. dorsal disc and arms; B. arm dorsolaterally; C. isolated lateral arm plate with spine articulations; D, E. arm vertebrae dissected from the inside of the arm; D. distal face; E. proximal face; F. ventral aspect of disc and arms; G. detail of jaw; H. dental plate from tip of jaw; I, J. oral plates(half-jaws); I. abradial face; J. adradial face. AS, adoral shield; ASS, adoral shield spine (often described as oral tentacle scale); CPP, central primary plate, DAP, dorsal arm plate; M, madreporite, OS, oral shield, RPP, radial primary plate; RS, radial shield; SA, spine articulation; TS, tentacle scale; VAP, ventral arm plate. Scale bars in millimetres.

At first glance, ophiuroids may resemble certain seastars, but a number of unique features set them apart. The ambulacral groove, found on the underside of the arms, is completely closed over by hard skeletal parts (lateral and ventral plates; Fig. 3), whereas in asteroids it is an open furrow. Ophiuroids lack an anus and the madreporite that connects the water vascular system (often through one or several hydropores) with the surrounding ocean water is part of the mouth skeleton (one of the oral shields), instead of a plate on the dorsal surface as in asteroids. The ophiuroid mouth opening is closed by a number of jaws that corresponds to the number of arms. The jaws or oral plates (Fig. 3I, J) are hypothesised to have evolved from ambulacral plates and are homologous to another ophiuroid specialisation, the arm vertebrae (Fig. 3D, E) [7]. Ophiuroid tube feet lack suction cups and are rarely used for locomotion. Instead, ophiuroids move by twisting and coiling their arms, pushing against the surface like a snake or gripping objects and pulling themselves forward. Swimming has been reported in some species [8]. No eyes have been found in ophiuroids, but arm plates, functioning as calcitic microlenses above light sensitive tissues have been identified in several phototactic species in the genus *Ophiocoma* [9]. Brittle stars easily fragment (autotomize arms) when stressed (Stöhr & O'Hara, personal observations), a property of the mutable collagenous tissue [10], found in all echinoderms.

**Figure 4:**
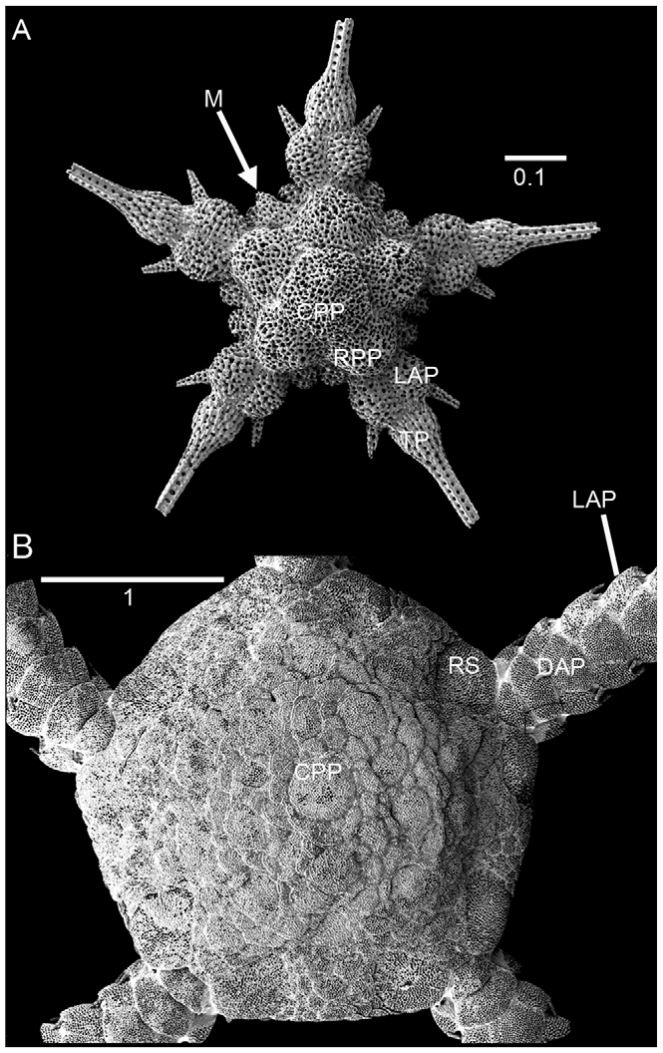
Comparison of juvenile and adult morphology. *Ophiopleura borealis*. SEM images. A. early postlarva, lacking dorsal arm plates, interradial disc scales and radial shields, madreporite lateral; B. small (young) adult, scales partially obscured by thickened skin. LAP, lateral arm plate; TP, terminal plate. Scale bars in millimetres.

For centuries, ophiuroid species were delimited and identified mainly on external adult characters. Recent efforts to describe juvenile characters have provided valuable new information (Fig. 4), but juvenile stages are still only known for less than 50 species [11], [12]. Promising results have been obtained by the inclusion of internal skeletal characters such as jaws and dental plates [13]–[15]. A limited number of molecular studies have been published so far, dealing mostly with problems of morphologically similar (cryptic) species [16]–[18]. The small number of genetic studies compared to other echinoderm groups is partly due to difficulties with efficiently obtaining suitable DNA sequences, but recent attempts have been made to solve these problems [19].

### Glossary

**Figure 5:**
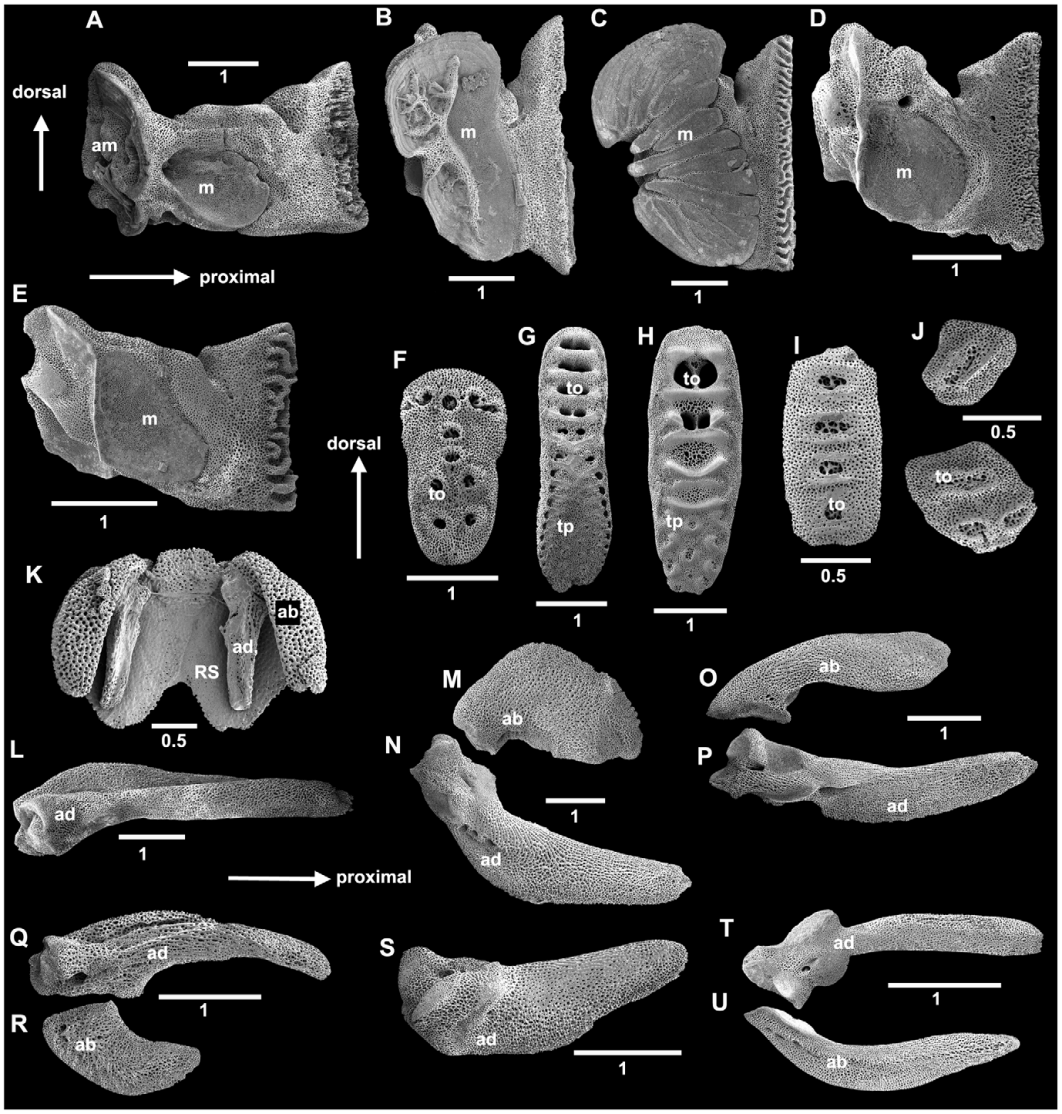
Diversity of ophiuroid skeletal elements: jaws, dental and genital plates. A–E. oral plates (half-jaws), abradial face; A. *Ophiura sarsii* (Ophiuridae), strongly elongated; B. *Ophiothrix fragilis* (Ophiotrichidae), short jaw with branch-like ornamentation; C. *Ophiocoma erinaceus* (Ophiocomidae), short jaw with striations; D. *Ophiacantha bidentata* (Ophiacanthidae), weakly elongated; E, *Ophioderma longicauda* (Ophiodermatidae), strongly elongated. F–J. dental plates, external (proximal) face; F, *O. sarsii*, multiple openings per tooth; G. *O. fragilis*, different areas for tooth papillae and teeth; H. *O. erinaceus*, different areas for tooth papillae and teeth; I. *O. bidentata;* J, *O. longicauda*, dental plate consists of several pieces; K–U. genital plates. K–L. *O. sarsii*; K, genital plates articulating with radial shields; M–N. *O. fragilis*; O–P. *O. erinaceus*; Q–R. *O. bidentata*; S, *O. longicauda*; T–U. *Amphiura chiajei* (Amphiuridae). ad, adradial; ab, abradial; am, articulation to arm; m, muscle attachment area; RS, radial shield.; to, tooth socket; tp, tooth papillae area; Scale bars in millimetres.

**Figure 6:**
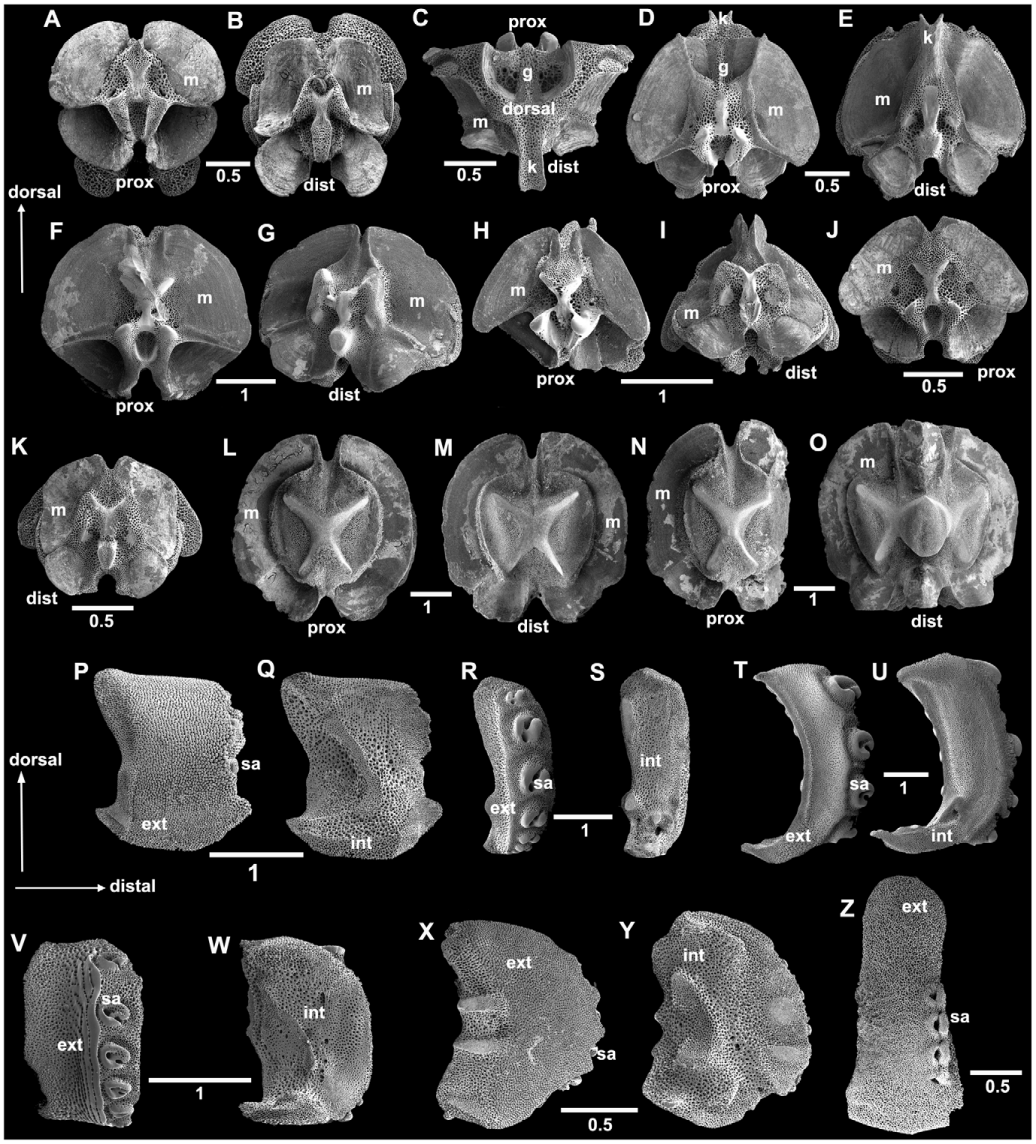
Diversity of ophiuroid skeletal elements: lateral arm plates and vertebrae. A–O. arm vertebrae; A–K. zygospondylous articulation. A–B. *Ophiura sarsii* (Ophiuridae); C–E. *Ophiothrix fragilis* (Ophiotrichidae), keeled type; F–G. *Ophiocoma erinaceus* (Ophiocomidae); H–I. *Ophiacantha bidentata* (Ophiacanthidae); J–K. *Ophioderma longicauda* (Ophiodermatidae); L–O. streptospondylous articulation, *Gorgonocephalus eucnemis* (Gorgonocephalidae); L–M. regular vertebrae; N. first vertebra of a new branch; O. last vertebra before a new branch (N and another similar vertebra articulate with O). P–Z. lateral arm plates. P–Q. *O. sarsii*; R–S. *O. fragilis*; T–U. *O. erinaceus*; V–W. *O. bidentata*; X–Y. *O. longicauda*; Z. *G. eucnemis*, dist, distal; ext, external; g, groove; int, internal; k, keel; m, muscle attachment area; prox, proximal; sa, spine articulation. Scale bars in millimetres.

The terminology used over the centuries for ophiuroid features has varied greatly between authors, which is a source of confusion, particularly for novices and non-specialists. The terms used for ophiuroid structures differ considerably from those used for other echinoderm classes, which contributes to the confusion. No official consensus has been reached yet, but more and more ophiuroid workers attempt to use the same terminology. As a step towards easier communication and understanding we propose here an illustrated glossary of terms that have been used most frequently in recent years. Figure 3 provides an overview over general ophiuroid anatomy with isolated skeletal elements and their position in situ. Figures 5 and 6 provide details of the structures described below.

**Aboral:** surface of the animal opposite the mouth, more often **dorsal** is used.

**Abradial (adj.):** away from central line of the arm.

**Accessory dorsal arm plate:** small plate on the periphery of the dorsal arm plate, found in Ophionereididae and *Ophiolepis*, not to be confused with fragmented arm plates found in e.g. *Sigsbeia*, *Ophioderma*.

**Adoral shield:** skeletal element, in pairs distal to oral shield, often separating it from the **oral plate**; homologous to lateral arm plate.

**Adradial (adj.):** close to the arm.

**Apical papilla(e):** oral papilla at tip of jaw, often homologous to first tooth; may be single or in a cluster.

**Arm:** moveable ambulacral projection attached to the disc, divided into segments (joints); the segments closest to the disc are the oldest, those at the tip of the arm the youngest.

**Arm comb:** row of papillae on the distal end of the abradial genital plate, next to either side of an arm base; only in the Ophiuridae.

**Arm spine articulation:** specific structures on lateral arm plates for attachment of spines; character of high taxonomic value, with family- and sometimes genus-specific shape (Fig. 3C, 5P, R, V, X, Z).

**Buccal scale:** distalmost lateral oral papilla, wide and low, at the oral plate; one of the first oral papillae in postlarvae (often modified during later ontogeny) of all examined Ophiuridae and Amphiuridae, moves higher up on the oral plate in *Amphiura* (Fig. 3G); as far as known absent in the Ophiotrichidae, Ophiomyxidae, Ophiocomidae and among ophiactids in *Ophiopholis aculeata* (Linnaeus 1767) (but present in *Ophiactis* spp.), in Ophiacanthidae so far found only in *Ophiolimna bairdi* (Lyman, 1883).

**Bursa(e):** sac usually on either side of an arm, holds the gonads, also respiratory function.

**Dental plate:** vertical plate covering the tip of each jaw, bearing teeth and apical papillae, often with holes and socket-like depressions (Fig. 4F–J, H).

**Disc:** central body, containing the main internal organs (Fig. 3A).

**Disc diameter:** common unit of size for ophiuroids, measured from the distal edge of the **radial shields** to the edge of the opposite **interradial**.

**Distal (adj).:** away from the disc center.

**Dorsal:** surface away from the mouth, more commonly used than **aboral**.

**Dorsal arm plate:** skeletal element on the dorsal part of each arm segment (Fig. 3B).

**Fissiparity:** asexual reproduction by division, here splitting of the disc, after which both halves regenerate a complete individual; common in hexamerous (six-armed) species.

**Genital papillae:** granule-like skeletal elements along the bursal slit; in Ophiuridae, Ophiochitonidae and Ophionereididae; in some genera (e.g. *Ophiura*) their row is elongated near the dorsolateral surface of the disc to form an **arm comb**.

**Genital plates:** a pair of skeletal elements to either side of each arm, supporting the bursal slit, articulating with each other distally and internally with the radial shield (Fig. 4K); the pair of plates are known as the **adradial** and **abradial** genital plate; they may be elongated, club-like, short, scale-like, forked or other (Fig. 4L–U).

**Genital slit (bursal slit):** external opening to the bursa.

**Granules:** articulated, or loosely attached, grain-shaped skeletal elements, may rub off, occur on disc and arms.

**Infradental papillae:** pair of **oral papillae** that originate laterally on the **dental plate** and then move onto the **oral plate**; only in Amphiuridae (Fig. 3G).

**Interradius (interradii):** the areas of the disc between the arms.

**Lateral arm plate:** plates on both sides of each arm segment, with a series of articulations bearing the arm spines (Fig. 3C); with family- and in some cases genus-specific characters, such as the presence and shape of excavations for the **tentacle pore**, elevations and holes, striations and elevated spine bearing ridges.

**Madreporite:** part of the ambulacral system, see oral shield.

**Oral:** side of the mouth, often termed **ventral** instead; also as adjective for structures in close association with the mouth.

**Oral papillae:** articulated skeletal elements along the jaw edges, may be spine-shaped, block-like, scale-like or other. Often distinguished as lateral papillae, along each side of a jaw, and one or several **apical papillae**, at the proximal tip of the jaw; absent in Ophiotrichidae; fused/not fragmented in some species of Ophiolepididae.

**Oral plate:** one half of a jaw (Fig. 3I, J, 4A–E), composed of a proximal and a distal part, sometimes with visible suture line, distal part with t**entacle pore** and **tentacle**, sometimes with **tentacle scale**.

**Oral shield:** large plate distal to each jaw, separated from the jaw by a pair of **adoral plates**, at least one oral shield functions as madreporite, often enlarged and/or with a visible hydropore.

**Ossicle:** see skeletal elements.

**Peristomial plates:** thin plates covering the dorsal (inner) surface of the oral frame.

**Plates:** larger, flat skeletal elements with fixed position (but the term plate is used as a more general term for skeletal element as well).

**Primary plates:** the central plates of the dorsal disc, composed of the central primary plate, surrounded by five radial primary plates (Fig. 3A), which together are also known as the primary rosette; present in most, but not all species, in adults not always distinguishable, in **postlarvae** they are the first plates that form the disc.

**Primary rosette:** see primary plates.

**Proximal (adj.):** towards the disc center.

**Radial shields:** pair of dorsal disc plates at the arm base (Fig. 3A), with internal distal articulation with the genital plates (Fig. 4K).

**Radius (radii):** the arms and areas of the disc where the arms are attached.

**Scales:** smaller, thinner, often more or less round skeletal elements, usually found on the disc, sometimes on the arms.

**Skeletal elements (plates, ossicles):** hard structures consisting of a Mg calcite meshwork, grown inside dermal cells; includes plates, scales, spines, granules, and papillae.

**Spines:** articulated, moveable skeletal elements of elongated shape, smooth or serrated, with terminal thorns or without, at arms and on disc; often distinguished as spinelets (smaller disc spines), stumps (short, blunt, usually thorny disc spines), spines (longer, rodlike, tapering, with or without thorns, at arms and on disc) and hooks, although these terms are not well defined. Arm spines are modified into hooks in epizoic species, sometimes only in juveniles or only at the distal arm segments. Bands of girdle hooklets occur on the dorso-lateral surface of Gorgonocephalidae arms.

**Stereom:** mesh-like structure of skeletal elements.

**Streptospondylous:** see vertebra.

**Stomach and gonad ossicles:** small rod-like, plate-like or ‘c’-shaped ossicles lining the walls of the stomach and gonads.

**Teeth:** small skeletal elements at the dental plate, block-like or spine-like (Fig. 3G).

**Tentacle:** tube foot.

**Tentacle pore:** opening on ventral arm, between **lateral** and ventral plate or as a perforation within the lateral arm plate, from which a tube foot protrudes, a pair of pores per segment.

**Tentacle rods:** small elongated ossicles strengthening the tube feet in the Ophiomyxidae.

**Tentacle scale:** articulated skeletal element at tentacle pore, may be at lateral arm plate and/or ventral arm plate, single or several, spine-shaped, scale-like or other (Fig. 3F).

**Terminal plate:** the last segment at the tip of an arm, tube-like, hollow; the terminal plate is present from the earliest postlarva to the largest adults, the arm grows by forming new segments proximal of the terminal plate.

**Tooth papillae:** cluster of short, granule-like apical papillae on the **dental plate**; in Ophiotrichidae and Ophiocomidae. Not to be confused with the cluster of larger, pointed apical papillae in some Ophiacanthidae.

**Tubercles:** non-articulated outgrowths of plates and scales, cannot be rubbed off (compare **granules**).

**Ventral:** side of the mouth, more commonly used than **oral**.

**Ventral arm plate:** plate on ventral side of each arm segment (Fig. 3F).

**Vertebra(e):** inner arm ossicle, one in each segment, composed from two ambulacral plates, often with visible suture line, which may separate during maceration; with distal and proximal articulations, traditionally classified as streptospondylous (hourglass-shaped) and zygospondylous, but intermediate types exist (Fig. 5A–O). Euryalida possess only streptospondylous vertebrae, in Ophiurida a variety of both streptospondylous and zygospondylous types occur. In Ophionereididae, Ophitrichidae and among Ophiactidae only the genus *Ophiopholis* the vertebrae have a dorsal keel, extending distalwards into a large groove on the proximal face of the following vertebra. Some vertebrae in the Euryalidae have a ventral bridge between the proximolateral processes that protects the radial canal and nerve.

**Zygospondylus:** see vertebra.

### Feeding

The ophiuroid digestive system is comparatively simple, consisting of a short oesophagus and a sac-like stomach with ciliated epithelium [20]. Lacking an anus, ophiuroids are not well equipped to extract nutrients from large amounts of ingested mud, in the manner of holothuroids or some asteroids and echinoids. Instead, ophiuroids display a broad range of feeding types, such as suspension-feeding, deposit-feeding, scavenging and predation, all designed for a more selective nutrient intake. Some species may use more than one feeding strategy, and diet as well as feeding type may vary between ontogenetic stages. However, few studies on the diet of ophiuroids have been conducted so far. Correlating feeding mode with taxonomic level (genus, family) is problematic also, since the systematics of ophiuroids is currently in flux (see below). Basket stars feed on plankton (copepods, apendicularians), clinging to sea pens or corals, using their often multi-branched arms to capture prey. Several species in the family Ophiuridae are carnivorous: *Ophiura ophiura* Linnaeus, 1758 hunts epibenthic animals, whereas *Ophiura albida* Forbes, 1839 and *Ophiura sarsii* Lütken, 1855 can hunt infaunal prey, scavenge carrion or feed off seafloor organic matter [21] and the Antarctic *Ophiosparte gigas* Koehler, 1922 is known to be an active predator of at least 10 phyla [22]. *Ophionereis reticulata* (Say, 1825) (Ophionereididae) is omnivorous, consuming both plant (algae) and animal material (polychaetes), as well as sediment, possibly scavenging or deposit feeding [23], amphiurids typically live in burrows, extending some of their arms above the sediment surface, collecting food from the burrow walls, the sediment surface and the water column with their tube feet [24], but the stomach content of *Amphipholis squamata* (Delle Chiaje, 1828) included fine particles as well as a wide range of animal and plant fragments indicating an omnivorous habit [25].

### Reproduction

A detailed review of ophiuroid reproduction was provided by Hendler [26]. In most ophiuroids, the gonads are restricted to the disc, although there are a few taxa (*Ophiocanops*, Euryalinae, Asteroschematinae) in which these organs extend into the base of the arms. The majority of ophiuroid species are dioecious, but hermaphrodites exist and self-fertilization has been shown for at least one species, *Amphipholis squamata* [27]. Males and females in most species look alike, but in *Ophiodaphne formata* (Koehler, 1905), *Ophiodaphne scripta* Mortensen, 1933, *Ophiosphaera insignis* Brock, 1888 and *Astrochlamys bruneus* Koehler, 1911 the male is much smaller than the female, to which it clings. In *Amphipholis linopneusti* Stöhr, 2001, both sexes are about the same size, but the males have an enlarged first ventral arm spine, hook-shaped in juveniles (perhaps facilitating attachment to their sea urchin host), wide and blunt in adults [28]. Many species are broadcast spawners that freely release their eggs into the water, others are brooders that keep the eggs, larvae and small juveniles inside the gonadal chambers (bursae) of their disc (e.g. *A*, *squamata*) or in the gonads (e.g. *Ophiacantha anomala* G.O. Sars, 1872) [26]. Asexual reproduction by fission, in which the disc splits into two halves, followed by regeneration, is common in hexamerous species such as *Ophiactis savignyi* (Müller & Troschel, 1842), although not all six-armed species are fissiparous. Brooding does not co-occur with fissiparity, for example the brooding six-armed *Ophiacantha anomala* does not divide [12]. Fissiparity in combination with hexamery is particularly common in the genus *Ophiactis* with so far 16 fissiparous six-armed species, but has been found in almost all families and many different genera. Likewise, brooding has been found in most families and continues to be discovered, sometimes in well-known species such as some populations of *Ophioderma longicauda* (Bruzelius, 1805) [29].

It is generally assumed that the ancient larval type of ophiuroids is the planktotrophic pluteus larva, but non-feeding plutei with abbreviated development and direct developing vitellaria larvae, are known as well [26]. It has been suggested that the presence of vitellaria larvae may facilitate the evolution of brooding [26] and in at least one species, *Ophioderma longicauda*, this appears to be a likely explanation [29].

### Life-style and habitat

Ophiuroids have adapted to a wide variety of life-styles. The majority of species are bottom dwellers on the sea floor, buried in mud or hidden in crevices and holes in rock or coral. Some species are epizoic, living on a variety of hosts such as gorgonian or black corals (many basket and snake stars, some Ophiotrichidae and Ophiacanthidae), sea urchins (e.g. *Amphipholis linopneusti*; *Ophiodaphne scripta* [28], [30], crinoids (e.g. *Ophiolophus novarae* Marktanner-Turneretscher, 1887, *Ophiomaza cacaotica* Lyman, 1871) [31], [32] or jellyfish (*Ophiocnemis marmorata* (Lamarck, 1816)) [33]. *Ophiactis savignyi* is a well-known sponge-dweller [34]. Juveniles of *Ophiomastix annulosa* (Lamarck, 1816) seek out adults of *Ophiocoma scolopendrina* (Lamarck,1816) and crawl into their bursae, where they live through the earliest stages of their development, similar to species that brood their young [35]. Some of these associations are of ancient origin. Most notable are cases of Jurassic and Cretaceous ophiacanthid brittle stars displaying the anatomical prerequisites for climbing and clinging (e. g. vertically coiling arms, hook-shaped spines) and found preserved as articulated specimens in close relationship with stalked crinoids [36], [37]. Articulated specimens of the small Middle Jurassic species *Ophiomusium*? *ferrugineum* Böhm, 1889 are commonly found in the dense isocrinid aggregations of the Burgundy platform [38], mostly preserved close to the proximal portions of the crinoid stalk. Another remarkable case of ophiuroid-host interaction known from the fossil record is the Late Palaeozoic genus *Onychaster*, articulated specimens of which have been reported tightly wrapped around stalked crinoids [39].

Brittle stars have been found at hydrothermal vents (*Ophioctenella acies* Tyler *et al*. 1995, *Spinophiura jolliveti* Stöhr & Segonzac, 2006 and *Ophiolamina eprae* Stöhr & Segonzac, 2006) [40], [41], methane cold seeps (*O. acies*, *Ophienigma spinilimbatum* Stöhr & Segonzac, 2005) [42] and on sunken wood (*Ophiambix* spp.) [43]. These species appear to be restricted to reducing environments and all, except *O. acies*, occur in only one type of environment.

Ophiuroids often occur in large numbers, sometimes in dense aggregations, such as *Ophiothrix fragilis* (Abildgaard, in O.F. Müller, 1789) in the British Sea [44], [45].

### Phylogeny

The so far only quantitative phylogenetic reconstruction of the Ophiuroidea has been performed by Smith *et al*. [46]. Their tree suggested that the family Ophiacanthidae is paraphyletic, because some of its species show close affinities to Ophiomyxidae and Hemieuryalidae and some of the species included in those families may better be placed in Ophiacanthidae. A recent new approach using the spine articulation on the lateral arm plates and internal skeletal characters suggested major changes in the ophiuroid phylogeny and proposed a clearer delineation of Ophiacanthidae from Ophiomyxidae [47], [48]. Ophiuroid higher taxa are difficult to delimit, because the class radiated over a relatively short time in the Late Paleozoic and Early Mesozoic, in particular after the mass extinction at the end of the Permian, and many species show character combinations that overlap with the diagnoses of several families. Our understanding of these characters and the selection pressures acting on them is still quite limited, but several projects are currently being executed in different workgroups to improve the situation.

The current phylogeny divides the Ophiuroidea into two sister groups, Euryalida (basket and snake stars) and Ophiurida (brittle stars) [46], [49], but the known fossil evidence does not support an early origin of the Euryalida [46]. Recent molecular evidence instead places it within Ophiurida [50], but more data are needed to confirm this hypothesis. Within the Euryalida, the Gorgonocephalidae have recently been confirmed as sister taxon to a clade consisting of Asteronychidae and Euryalidae [51].

### Fossil record

The skeleton of brittle stars is composed of high-Mg calcite which is transformed into low-Mg calcite during diagenesis. Thanks to the high chemophysical stability of low-Mg calcite and the transformation occurring early in the process of fossilisation, the ophiuroid skeleton is likely to be preserved in most types of marine rocks. From a taphonomical point of view, however, the ophiuroid skeleton is composed of a multitude of plates connected by soft tissue and disintegrates within hours to days after death [52]. Articulated specimens with most of the skeletal plates in place are extremely rare fossils (Fig. 6) and document exceptional cases of rapid and definite burial preventing dislocation of the skeletal plates [53]. Dissociated plates of the ophiuroid skeleton, in contrast, occur in large amounts in most marine sediment and can account for a considerable portion of micropalaeontological samples.

Ophiuroids have been shown to display a remarkable morphological conservatism, at least since the early Mesozoic. Yet, many known fossil taxa have not been studied from the perspective of modern representatives. The global diversity of ophiuroids in the course of the Phanerozoic is also still poorly understood. The number of currently accepted brittle-star species from the Jurassic, one of the best sampled geological time intervals, is approximately 70 (Thuy, unpublished data), and even acknowledging a limitation of the Jurassic ophiuroid fossil record to shallow-water settings, this is an extremely low total diversity for a time interval spanning more than 40 Ma compared to present-day diversity. The low diversity of fossil brittle stars is clearly due to a lack of systematic sampling. Most records of fossil ophiuroids represent occasional findings of articulated specimens. It has been repeatedly demonstrated that species diversity can dramatically increase as soon as the diagnostic skeletal elements preserved as microfossils after disintegration are taken into account [36], [54], [55]. The inclusion of dissociated skeletal plates in the survey of fossil ophiuroids is highly promising, but still a poorly deployed perspective, in particular when combined with detailed morphological studies of the respective skeletal parts in recent ophiuroids [5], [47].

The oldest currently known ophiuroid is *Pradesura jacobi* (Thoral, 1935) from the Late Tremadocian (Early Ordovician, ∼480 Ma) of southern France [56]. It belongs to an extinct group of ophiuroids displaying plesiomorphic characters not found among extant adult forms, the most conspicuous being the unfused ambulacral plates (pairs are firmly fused into vertebrae in extant ophiuroids). These assumed stem-group ophiuroid representatives were fairly diverse during the Ordovician and Silurian [57]), but by the Late Carboniferous they had nearly disappeared and were outnumbered by groups with closer affinities to modern ophiuroids.

Less than one third of the extant ophiuroid families are known from the Early Mesozoic and include the Ophiacanthidae, Ophiuridae and Ophiolepididae [5], [37]. The majority of the families which dominate present-day shallow tropical and temperate habitats, in particular the Ophiocomidae, Ophiotrichidae, Amphiuridae and Ophiactidae, seem to be of Late Mesozoic origin [36], [58], thus challenging the major radiation of modern ophiuroid clades in the Early Triassic as postulated by Smith *et al*. [46]. In addition, Upper Devonian to Lower Carboniferous ophiuroids were recently demonstrated to have strong affinities with extant ophiolepidid brittle stars, suggesting that at least part of the crown-group radiation took place much earlier than previously assumed [59]. A reassessment of Upper Paleozoic and Lower Triassic ophiuroids in close comparison with modern clades is required to further elucidate the early evolution of the crown-group ophiuroids. Many post-Paleozoic ophiuroid taxa are incompatible with family concepts of extant ophiuroids (e.g. [37]). This has lead to the recognition of new, extinct families (e.g. Aplocomidae by Hess, 1965, [60]) which potentially contribute to a better understanding of the origin of and phylogenetic interrelationships among extant lineages.

## Methods

Ophiuroid species names were collected from the literature and entered into the online World Ophiuroidea Database [1], part of the World Register of Marine Species (WoRMS) [6]. The current taxonomic status of the about 3000 nominal species and over 4000 names (including new combinations) was assessed and recorded in the database. Then these data were used to assemble Table 1, numbers of species and genera per family. The systematics largely follows Smith *et al*. [46], except where more recent information is available. Ophiocanopidae was removed by Stöhr *et al*. [61] and its only genus *Ophiocanops* is included in Ophiomyxidae. The genera *Ophiomoeris* and *Ophiochondrus*, formerly placed in Hemieuryalidae, have recently been transferred to Ophiacanthidae [47]. The systematics of the Euryalida has been revised recently and the family Asteroschematidae has been lowered to subfamilial rank within Euryalidae [51].

**Table 1:** Species diversity of extant Ophiuroidea, derived from the online “World Ophiuroidea database”, excluding subspecies.

Order	Family	Genera	Species described
Euryalida	Asteronychidae	3	9
	Euryalidae	10	77
	Gorgonocephalidae	34	95
Ophiurida	Amphilepididae	1	14
	Amphiuridae	34	467
	Hemieuryalidae	7	10
	Ophiacanthidae	35	319
	Ophiactidae	5	69
	Ophiochitonidae	2	18
	Ophiocomidae	8	78
	Ophiodermatidae	21	109
	Ophiolepididae	16	164
	Ophiomyxidae	29	88
	Ophionereididae	5	34
	Ophiotrichidae	16	169
	Ophiuridae	44	344
Total		270	2064

A biogeographic analysis of the world's extant ophiuroid species was performed by extracting a list of described species from the World Ophiuroidea Database [1]. Distributional data was obtained from a global database of museum catalogue sample data [62], supplemented by additional records from the taxonomic literature to ensure a coverage of all species. We selected this database, because the World Ophiuroidea Database is complete with regard to taxonomic information, but still lacking in distributional data. Other possible databases that collect distribution data are the Encyclopedia of Life (EoL), the Global Biodiversity Information Facility (GBIF) and the Ocean Biogeographic Information System (OBIS), but none of these has yet sufficient amounts of data. The imprecise nature of the data contained in older taxonomic literature did not permit a quantitative approach to defining biogeographical regions. Instead, the world's marine environment was divided into 12 *a priori* large-scale regions based on available information (Figure 6, see below) and four depth strata: shelf (0–200 m), bathyal (200–3500 m), abyssal (3500–6500 m) and hadal (below 6500 m) [63]. The aerial extent of these regions and depth strata was calculated from the ETOPO bathymetric dataset [64]. Equatorial regions were defined as being bounded by the 30° latitude in both hemispheres, the approximate boundary of tropical shallow-water coral-reef distributions [65] and the bathyal tropical-temperate transition in the Indo-Pacific [46], [59]. Polar regions were bounded by 60° latitudes, thus separating the Antarctic continent from most of the subantarctic islands [66]. Temperate/boreal regions were defined as falling between these zones, 30–60° in each hemisphere. Longitudinal boundaries were set for the equatorial and southern temperate regions in mid-ocean reflecting the faunal relationship between offshore areas and nearby continental margins. The Indian Ocean boundary was set at 90°E, placing the Chagos and St Paul/Amsterdam islands in the Indian and South Africa regions respectively, and the Christmas/Cocos Islands and Indo-Malay archipelago in the Indo-Pacific region. The Atlantic regions were broadly separated by the Mid-Atlantic Ridge. The boundary in the Pacific Ocean was placed between the eastern Pacific islands of Juan Fernandez-Galapagos-Clipperton and the Indo-Pacific Hawaii-Pitcairn-Easter Islands. These regions reflect our knowledge of the fauna at shelf and upper bathyal depths, however, we have adopted the same regions for deeper areas to facilitate inter-depth comparisons. In reality, species ranges will not be exactly congruent and adjacent biogeographic regions or depth strata are likely to form broad transition zones, making it problematic to define precise biogeographical boundaries [62]. The temperate regions in particular contain enhanced species turnover between tropical, temperate and polar faunas [62]. The lack of quantitative location data from the older taxonomic literature also precludes the adjustment of regional species richness by sampling effort [67]. Despite these limitations, we believe that the data are useful for a first approximation of global ophiuroid biogeography.

## Results and Discussion

### Species diversity

Evaluating global ophiuroid diversity is difficult, because many species have not been reported again since their original description and their current taxonomic status is unknown. The scientific effort has varied over the centuries, resulting in patchy knowledge, and brittle stars have received comparatively little attention during the past 50 years. Species inventories are more reliable for better known areas such as the North Atlantic, although, even here they are far from complete, as the discovery of ten new species in the North Atlantic since 2003 shows [42], [68], [69]. Published records for less well known areas, such as the Pacific Ocean, require careful analysis and verification, as many species have been described more than once and need to be revised [70]. The species list presented in WoRMS has been accumulated from publications, but many of the species names have never been revised. Consequently, the precise number of species and their taxonomic status change as new information is gathered.

The extant Ophiuroidea are currently divided into two orders and 16 families; the largest are Amphiuridae (467 species), Ophiacanthidae (319 species) and Ophiuridae (344 species), and the majority of the species (1883) belong to the order Ophiurida (Table 1). Species in the genera *Ophiothrix* and *Macrophiothrix* (family Ophiotrichidae), abundant in shallow tropical habitats, are morphologically similar and difficult to identify. Morphological and molecular evidence suggests that their species diversity is currently underestimated. Approximately 260 undescribed species from various families have been putatively identified to date (O'Hara unpublished data) and there are possibly several hundred more remaining to be identified (Stöhr & O'Hara unpublished data). Moreover, with the increase in molecular data, more cryptic species can be expected to be discovered [71].

### Biogeography

The 2064 described ophiuroid species are distributed from the intertidal to hadal depths, from the equator to polar regions (Table 2). Globally, there were approximately similar numbers of species recorded from the shelf (n = 1313) and bathyal depth strata (1297), although the total area of shelf (30.5 million km2) was only a third of that from bathyal depths (93.9 million km2). Only 109 species were recorded from abyssal depths despite the massive scale of the available habitat (240.2 million km2). Only 25 of these species were restricted to abyssal depths, another four occur in both abyssal and hadal habitats, and a further three were only recorded from hadal depths (2.2 million km2). These low numbers will almost certainly be boosted by further collection effort. Mollusc researchers have proposed that abyssal animals are often too sparsely distributed to maintain their own populations but instead are largely derived by dispersal from bathyal sources [72].

**Table 2:** Species richness and endemism of all described ophiuroids across 12 a priori defined regions and four depth strata.

			Number of species in each depth stratum					Area (million km2)				
Region	No of species in region	Species endemic to region (%)	Shelf (0–200 m)	Bathyal (200–3500 m)	Abyssal (3500–6500 m)	Hadal (>6500 m)	Unknown depth #	Shelf (0–200 m)	Bathyal (200–3500 m)	Abyssal (3500–6500 m)	Hadal (>6500 m)	Total
Arctic	73	8.2	36	60	7	0	0	6.9	8.9	1.6	0.0	17.4
North Atlantic	241	23.7	138	180	30	0	3	4.8	8.1	8.9	0.0	21.8
North Pacific	398	50.8	262	259	20	2	21	2.8	4.5	18.3	0.7	26.3
West Atlantic	335	60.6	217	229	16	0	3	2.2	4.5	11.0	0.2	18
East Atlantic	118	39.8	73	63	17	2	0	0.6	3.7	21.0	0.1	25.4
Indian	316	25.6	222	160	19	1	4	1.7	8.9	18.0	0.0	28.5
Indo-Pacific	825	47.5	551	507	31	6	6	6.8	21.2	70.3	1.0	99.3
East Pacific	186	62.9	92	111	28	1	4	0.4	6.0	13.5	0.0	19.9
South Africa	201	21.9	152	135	20	1	4	0.2	7.9	20.0	0.0	28.1
South Pacific	355	22.8	235	259	21	0	0	0.9	9.9	33.8	0.0	44.6
South America	124	24.2	79	102	17	1	1	1.5	2.8	11.1	0.2	15.6
Antarctic	126	36.5	72	105	27	1	5	1.6	7.4	12.8	0.0	21.8
Unknown #	9		1				8					
Total species *	2064		1313	1297	109	7	52					

The area of each region/depth strata was calculated from the ETOPO bathymetric dataset (Amante & Eakins 2008).

# A few species were described from specimens without known sample locality or depth information.

* As species can occur in more than one region and depth stratum, the total species counts are not a simple arithmetic sum of regional species richness.

Although shelf and bathyal habitats have similar numbers of species, there was generally a considerable difference between their constituent species [62]. In shallow water at tropical and temperate latitudes, assemblages were dominated by the families Ophiotrichidae, Ophionereididae, Ophiocomidae, Ophiodermatidae, Ophiactidae and Amphiuridae. Remaining families mostly occurred at deeper depths. There were some exceptions, for example *Bathypectinura* (Ophiodermatidae) occurred at bathyal depths [73] and there were some species of *Ophiacantha* (Ophiacanthidae) and *Ophiura* (Ophiuridae) in coastal zones. Some species appeared to be eurybathic, the diminutive *Amphipholis squamata*, as understood today, was found from the intertidal zone to 1200 m, but this species is likely comprised of a complex of several cryptic species [16], [17]. Polar species tended to be more eurybathic than temperate or tropical ones, with bathymetric ranges of shallow water Antarctic species frequently extending beyond 1000 m [62]. However, it was unclear whether this fauna was derived from an emergent bathyal fauna or vice-versa (cf [74]for octopodids).

**Figure 7:**
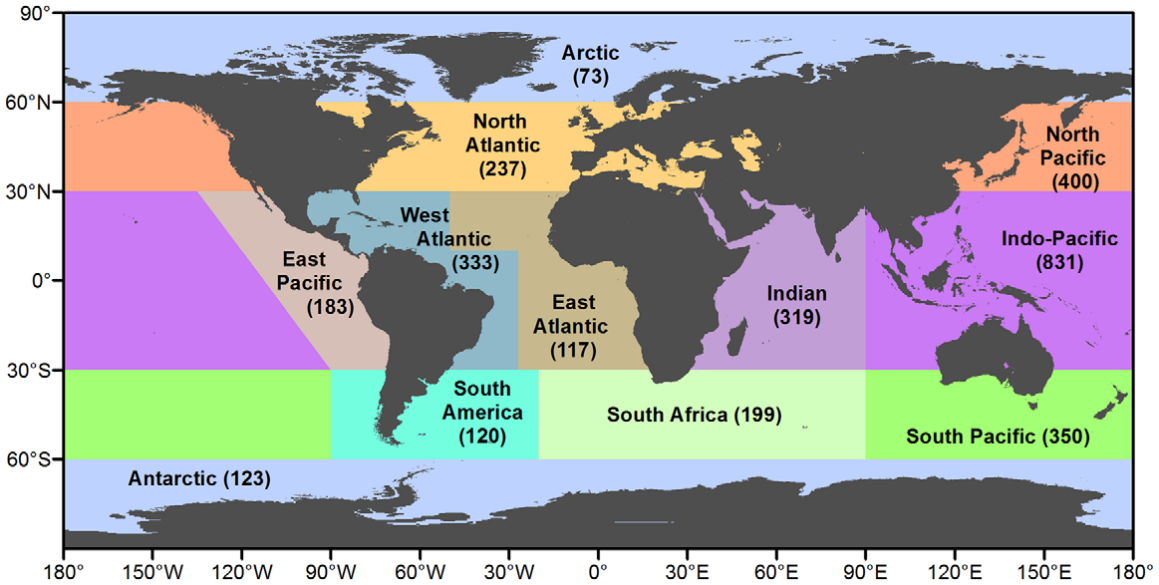
Global distribution of described species of Ophiuroidea, based on Table 2.

The Indo-Pacific region had the highest species richness overall (825 species) and at all depths (Table 2, Fig. 7). Adjacent regions were also relatively species rich, including the North Pacific (398), South Pacific (355) and Indian (316) due to the presence of many Indo-Pacific species that partially extended into these regions. The West Atlantic was a secondary region of enhanced species richness (335). Regions of relatively low species richness include the Arctic (73 species), East Atlantic (118), South America (124) and Antarctic (126). Some of the species richness of the Indo-Pacific could be attributed to its vast area (99.3 million km2). Sixty-four percent of species (1316) were restricted to a single region. The regions with the highest proportion of endemic species included the East Pacific (63%) and West Atlantic (61%), although this could be in part due to the lack of recent taxonomic reviews of the bathyal fauna (O'Hara, unpublished data). The lowest level of endemism was in the Arctic (8%), presumably reflecting the faunas relatively recent origin [75]. Antarctic in contrast had 37% endemism. Generally, the temperate regions have lower rates of endemism, due to the overlap of tropical/temperate and temperate/polar faunas; the exception is the North Pacific (51%).

A few species are widespread across the globe. At shelf depths, the viviparous *Amphipholis squamata* has been recorded from all regions except the poles. A few shelf species occur in all tropical regions, for example the abundant fissiparous species *Ophiactis savignyi*. However, molecular analyses suggest that both these species may consist of a suite of cryptic forms [16], [76]. Species ranges tend to be greater in the bathyal and abyssal zones, for example *Asteronyx loveni* Müller & Troschel, 1842, *Ophiura irrorata* (Lyman, 1878), *Ophiomusium lymani* Wyville-Thomson, 1873, *Ophiocten hastatum* Lyman, 1878 and *Amphiophiura bullata* (Wyville-Thomson, 1878) have been reported from across the Atlantic, Indian, Pacific and Southern Oceans, although again some of these species appear to have morphological variants [77] that need to be confirmed by modern molecular studies. Seamount faunas are also widespread at temperate latitudes, for example *Ophiactis abyssicola* (M. Sars, 1861) and *Ophiacantha spectabilis* G.O. Sars, 1871, often associated with cold-water corals [62].

Most differences between regional and intra-regional faunas tend to be at the species-level. All families and most genera are longitudinally widespread; there is little evidence for the long-term isolation of oceanic basins or seas [62]. Speciation processes are unclear, particularly at bathyal and abyssal depths. There are some cases where similar species appear to be segregated by depth (e.g. *Ophiacantha bidentata* (Bruzelius, 1805) and *O. fraterna* Verrill, 1885 in the Atlantic [69]; *Acrocnida brachiata* (Montagu, 1804)/*spatulispina* Stöhr & Muths, 2010 [15]). There are several shallow-water species separated by the Isthmus of Panama which has emerged over the past 2–19 million years [78], for example *Ophiocoma pumila* Lütken, 1856/*O. alexandri* Lyman, 1860 and *O. echinata* (Lamarck, 1816)/*O. aethiops* Lütken, 1859 [79]. Some genera have interesting anti-tropical distributions, for example *Ophiopteris papillosa* (Lyman, 1875) (California) and *O. antipodum* E.A. Smith, 1877 (New Zealand). However, these distributions may be relicts from former more widespread ranges. For example, fossils of the genus *Ophiocrossota*, currently restricted to southern Australia, have been found in Eocene and Miocene strata of North America [80], [81].

Global patterns of benthic species richness have been assembled for several other benthic groups including bivalves/gastropods [82], galatheids [83], stylasterids [84] and ascidians [85] Some patterns appear to be general; latitudinally, the poles have reduced species richness, and longitudinally the Pacific Ocean tends to be more speciose than the Atlantic. Other regional patterns are more taxon specific. The East Indo-West Pacific region is the peak of species richness for bivalves/gastropods and galatheids, whereas the South-West Pacific appears to be the peak for stylasterids and ascidians. Species richness in the eastern Pacific is high for bivalves and gastropods but low for galatheids, in South America it is relatively high for ascidians but also low for galatheids, in the northern Pacific it is high for bivalves and ascidians but low for gastropods and galatheids, and South Africa is very high for gastropods. It is unclear how much these patterns are biased by differences in spatial and bathymetric sampling effort and in regional definitions.

### Human interest

Ophiuroids are rarely harvested directly by humans, although some species of *Ophioderma* and *Ophiarachna* are sold as marine aquarium species (O'Hara, unpublished data). On the other hand, as they are a dominant component of seafloor faunas, they can be impacted by other human activities such as mining or trawling [86]. Scientifically, ophiuroids have emerged as a key taxonomic group for macro-ecological or biogeographic studies, because they occur in all marine habitats, have a range of trophic and life history strategies, and are diverse and abundant enough to statistically analyse without being so diverse that every survey becomes a major taxonomic exercise. From a palaeontological perspective, ophiuroids offer a high potential to act as model organisms for the assessment of macro-evolutionary patterns and the impact of palaeoceanographic events on the composition and diversity of past communities, because their skeletal parts are taxonomically identifiable and occur in great numbers as microfossils in most marine sediments, including deep-sea cores.

### Future research

Future biodiversity research must include additional molecular studies. We need a comprehensive phylogeny of the group, the lack of which is currently a major impediment to understanding ophiuroid biogeography and evolution. In many cases we do not understand species limits. Almost every molecular study on ophiuroids to date has resulted in the discovery of further cryptic species [71]. Conversely, bathyal and abyssal species may be more widespread than we think because regional variants have been described as separate species.
